# Anthelmintic Efficacy of Supramolecular Complex of Praziquantel Obtained by Mechanochemical Technology

**DOI:** 10.18502/ijpa.v15i3.4201

**Published:** 2020

**Authors:** Ivan ARKHIPOV, Salavat KHALIKOV, Alexander DUSHKIN, Konstantin SADOV, Elizaveta METELEVA, Michael ARISOV, Anastasiya VARLAMOVA

**Affiliations:** 1. Department of Experimental Therapy, All-Russian Scientific Research Institute of Fundamental and Applied Parasitology of Animals and Plants, Branch of Federal Scientific Center of Experimental Veterinary Medicine, Russian Academy of Sciences, Moscow, Russia; 2. Department of Physiologically Active Organofluorine Compounds, A.N. Nesmeyanov Institute of Organoelement Compounds, Russian Academy of Sciences, Moscow, Russia; 3. Department of Mechanochemistry of Organic Compounds, Institute of Solid State Chemistry and Mechanochemistry of the Siberian Branch, Russian Academy of Sciences, Novosibirsk, Russia

**Keywords:** Efficacy, Praziquantel, Supramolecular complex, Mechanochemistry, Disodium salt of glycyrrhizic acid

## Abstract

**Background:**

A supramolecular complex of praziquantel (PZQ) with disodium salt of glycyrrhizic acid (Na_2_GA) was obtained by mechanochemical technology to increase solubility, absorption rate and hence bioavailability of the drug and reduction its therapeutic doses. The aim of our study was evaluation of anthelmintic efficacy of supramolecular complex of PZQ.

**Methods:**

Different samples of PZQ with Na_2_GA were obtained by mechanochemical processing and examined for some physico-chemical properties. The anthelmintic activity of the most perspective samples was studied on the laboratory model of *Hymenolepis nana* infection of mice and *Moniezia expansa* infection of sheep by the results of helminthological necropsy of the small intestines (the controlled test).

**Results:**

A high efficacy (> 98%) of supramolecular complex of PZQ with Na_2_GA (1/10) was shown at doses of 3; 2 and 1 mg/kg of body weight at single oral administration against *H. nana* in mice and *M. expansa* in sheep. While the basic PZQ had 27.19% and 36.64% efficacy respectively at the dose of 1 mg/kg. The PZQ:Na_2_GA 1/10 physical mixture (without mechanochemical processing) revealed no anthelmintic efficacy.

**Conclusion:**

Joint mechanochemical treatment the PZQ substance and Na_2_GA led to increased solubility, reduction of particle sizes, amorphization of substance, incorporating it with micelles of glycyrrhizic acid and high anthelmintic efficacy in reduced dose. The supramolecular complex of praziquantel was found to be a perspective anthelminthic with enhanced pharmacological activity that needs further research.

## Introduction

Praziquantel (PZQ) is the main drug for treatment of cestodosis of animals and humans (1–3). However, high doses of PZQ (50–100 mg/kg) are required to treat larval cestodosis of animals that restrict its practical application (4, 5). The high cost of the drug, as well as its high consumption for treatment of large animals (cattle, horses), restrain its use. In this regard, there was an interest in reducing the dose of the drug by increasing its solubility, absorbability and efficacy using mechanochemical technology to produce solid dispersions of anthelmintics with hydrophilic carriers - plant and synthetic polymers, as well as with lipophilic-hydrophobic substances as drug delivery systems.

As a carrier, we have used disodium salt of glycyrrhizic acid (Na_2_GA). An important feature of glycyrrhizic acid (GA) solutions is the possibility of including lipophilic low-soluble molecules in the structure of micelles or other self-associates, that significantly increase the overall concentration of their molecules in a free and bound form in aqueous solutions (6, 7). Nevertheless, the use of pure GA as a carrier of drug molecules has a disadvantage in the phenomenon of gelling of its concentrated solutions. The use of its disubstituted salts of alkali metal makes it possible to eliminate this phenomenon.

Thus, the aim of our study was to obtain a supramolecular complex of PZQ with Na_2_GA by mechanochemical technology and to study its physicochemical features and anthelmintic activity in comparison with the substance of original PZQ (without Na_2_GA) and with the physical mixture of PZQ:Na_2_GA 1/10 without mechanochemical processing.

## Materials and Methods

The research was blinded, randomized and placebo-controlled. Studies were carried out according to the adopted rules (8–10). Council on Ethics at the Ministry of Health of Russia approved the trials. Clinical trials governmental registration protocol № 615–110; 25.09.2017.

Solid phase was conducted in one step of mechanochemical treatment of PZQ with Na_2_GA in a Ball Drum Mill LE-101 (Hungary) in a weight ratio of 1:5, 1:10 and 1:20 (11, 12).The drum was set on rolls and the mixture was processed for 4 h at a speed of 156 revolutions per minute; acceleration of grinding media - 1g with steel balls with diameter of 22 mm. Other samples were obtained by mechanochemical technology without an auxiliary substance - Na_2_GA by processing in planetary mill AGO-2 (Novosibirsk, Russia). Processing mode: acceleration of grinding media – 20 g and 40 g, weight of the material – 2.5 g, drum capacity – 50 mL, grinding media-steel balls (diameter 6 mm, 75.0 g load), processing time – 5 min (11, 12).

### Characterization

The solubility of samples was evaluated by dissolution (approx. 0.55 g) in distilled water (10 ml) in shaker-incubator at 37 °C and 200 rpm for three hours. The concentration of drug substances in solutions was analyzed by HPLC on Agilent 1200 (Agilent technologies, Santa Clara, USA) with a Zorbax Eclipse XDB-C18 column (50 × 4.6 mm) and diode-array detector; eluent: acetonitrile/acetate buffer pH 3.4 (1:2); detection on wavelength: 210 nm. The thermal analysis was conducted by Differential Scanning Calorimeter-550 (Instrument Scientific Specialists Inc., Twin Lakes, USA) in argon atmosphere (20–250 °C; rate of heating: 10 °C/min). The morphology of tested substances was studied by Hitachi microscope TM-1000 (Hitachi High-Technologies Corp., Tokyo, Japan). Diffractometer DRON-4 was used for X-ray analysis with CuK_α_-radiation at 2 deg/min (l=1000) in the angle range of 4–65 degrees (Bourevestnik Inc., St. Petersburg, Russia). The method of Higuchi and Connors was used for phase solubility studies (13). After equilibrium of dissolved samples was reached, the suspensions were centrifuged (12000 rpm; 10 min). Super-natants were filtered and examined for PZQ concentration by HPLC. The total molar concentration of Na_2_GA (Х-axis) and the total molar concentration of PZQ (Y-axis) were used for construction of the phase solubility diagram. In vitro parallel artificial membrane permeability assay (PAMPA) experiment was conducted in 12-well filter plates (polycarbonate membrane, 12 mm diameter, 0.4 μm pore size, 1.12 cm^2^ area, Corning Incorporated) to predict passive intestinal absorption (14). The artificial membrane was saturated by pipetting 60 μL of the 5% hexadecane in hexane solution to each wells of the donor plate. After hexane evaporation about 1.5 mL of distilled water was added to each wells of the acceptor plate. The hexadecane treated donor plate was then placed on top of the 12-well acceptor plate. Then, 0.5 mL of PZQ or its compositions solutions in distilled water were added to each well of the donor plate, and the plate was placed in shaker-incubator for 2.5 h (37 °C, 200 rpm). Samples (1 mL) were collected from the acceptor plate at appropriate time points (0.25h, 0.5 h, 0.75h, 1.0 h, 1.25h, 1.5 h, 1.75h, 2 h, 2.25h, 2.5h) and were analyzed by HPLC, and the same volume of distilled water was replenished. All these methods have been described previously (11, 12, 15–17).

### Study Animals

#### H. nana infection of mice

The study of the efficacy of supramolecular complex of PZQ with Na_2_GA (1:10) against *H. nana* infection of mice was conducted on 50 inbred white mice BALB/c (Branch Stolbovaya FSBES of NCBMT FMBA of Russia) of both sexes (16–18 g). Mice were quarantined and adapted for 7 days before the experiment. Then animals were divided into polycarbonate cages of 6 animals each in the vivarium with controlled environment of 20–22 °C and humidity of 60–70 %. Mice received rat feed (LLC Laboratorkorm, Moscow, Russia) according to the daily feed rate of the Russian Federation (18). Water was provided *ad libitum* during the study. Animals were randomly selected in groups by the method of random numbers and with the same body weight for the trials.

Cestodes of *H. nana* collected from the previous infection were destroyed in a small volume of tap water by repeatedly sucking into a syringe with a needle-cannula. About 200 infective eggs of *H. nana* were administered orally to each mouse with a disposable syringe of 1 ml connected to 25 mm needle. After infection - on the 5^th^ day after inoculation, the feces were examined daily by the sedimentation method in order to find eggs of *H. nana* (19).

#### Monieziosis of sheep

The anthelmintic activity of supramolecular complex of PZQ was studied in sheep farm of Samara region (LTD Agro resource), where high levels of *Moniezia expansa* and *Moniezia benedeni* infection have been registered. Experiments were conducted during the period of maximum infection on 60 young sheep of Stavropol Merino breed weighing from 17 to 32 kg in 2017. Sheep were kept indoors and fed according to the norms and rations of feeding livestock (20). Water was provided *ad libitum* during the experiment. Fecal examinations were made by the standard fecal examination techniques before the study for random distribution of sheep into the experimental groups with the same number of eggs per gram of feces (21).

#### Materials

PZQ (2-(Cyclohexylcarbonyl)-1,2,3,6,7-11bhexahydro-4*H*-pyrazino[2,1-*a*]isoquinolin-4-one), 98.5%, average Mw ~ 312.41 was purchased from Rensin Chemicals Limited (Nanjing, China). Na2GA was purchased from Shaanxi Sciphar Biotechnology Co. LTD (Xian, China). All other chemicals and solvents were of analytical reagent grade.

#### Experimental groups

One control group and 5 experimental groups of 10 animals were formed randomly on the 13^th^ day after infection by the results of feces examination by McMaster method (21). The supramolecular complex of PZQ with Na_2_GA was administered intragastrically to the mice of the 1–3 experimental groups with a stainless steel bulb tipped gavage needle in a dosage rate of 3.0; 2.0 and 1.0 mg/kg of active substance. The 4^th^ experimental group received basic PZQ at the dose of 1.0 mg/kg of b/w. One percent starch gel was given to the animals of control group. PZQ: Na_2_GA1/10 physical mixture was administered to the animals of the fifth group at the dose 2 mg/kg of active substance. The animals were sacrificed by cervical dislocation on the 4^th^ day after drugs administration.

Sheep naturally infected with monieziosis were divided into 5 equal groups of 10–12 animals each by the results of feces examination by McMaster technique (21). The supramolecular complex of PZQ with Na_2_GA was administered orally to the animals of the first, second and third groups in the form of 10 % powder once at doses of 1.0, 2.0 and 3.0 mg/kg of b/w respectively. Sheep of the 4^th^ received a basic drug – PZQ at the dose of 1.0 mg/kg. The animals of the control group received no preparation.

#### Procedures

The efficacy of the supramolecular complex of PZQ with Na_2_GA against *H. nana* infection of mice was evaluated on the 4^th^ day after drug administration by the results of helminthological necropsy of mice intestines. Recovered cestodes were counted. The anthelmintic activity was determined by the results of controlled test (22). Identification of *M. expansa* was based on the distinct morphology of helminthes by the results of helminthological necropsy of sheep intestines on the 10^th^ day after treatment (23).

### Statistical methods

Statistical analysis of data was performed on geometric mean of number of parasites using the parametric *t*-test to compare differences between treatment groups and control groups for significance at the *P* ≤ 0.05. Statistical analysis of data was evaluated by SAS/Stat (version №9.4 of the SAS System for Windows) software.

## Results

**Fig. 1:**
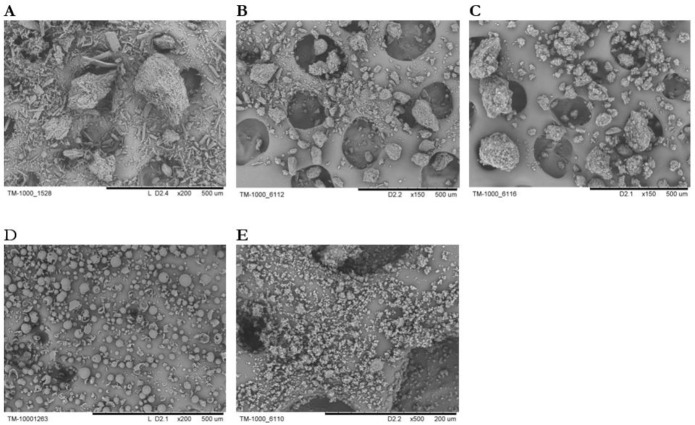
Electron micrographs of initial substance of praziquntel (A); B. praziquantel processed in AGO-2 mill (20g, 5 min.); C. praziquantel processed in AGO-2 mill (40g, 5 min.); D. Na_2_GA; E. praziquantel/Na_2_GA 1/10 processed in ball drum mill for 4 hours obtained by mechanochemical technology (1/10)

**Fig. 2:**
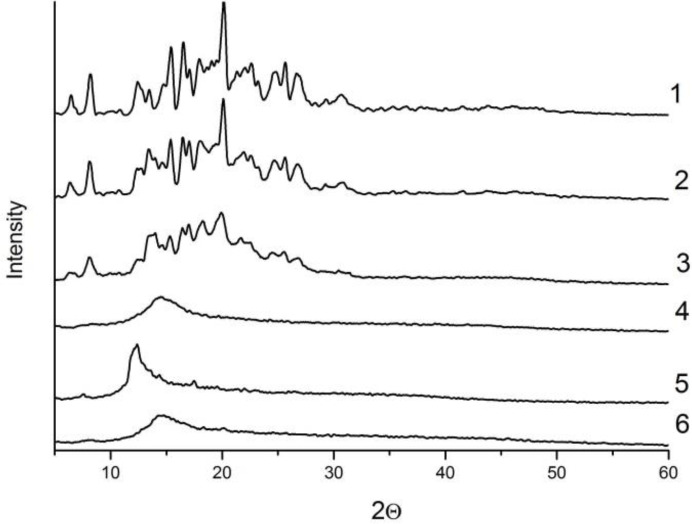
Radiographs of XRA of: 1. praziquantel; 2. praziquantel processed in AGO-2 mill (20g, 5 min.); 3. praziquantel processed in AGO-2 mill (40g, 5 min.); 4. Na_2_GA; 5. physical mixture of praziquantel/Na_2_GA 1/10; 6. praziquantel/Na_2_GA 1/10 processed in ball drum mill for 4 hours

The micrographs obtained by electronic scanning microscopy showed that the initial PZQ consists of crystalline elongated particles of 10–20 μm in size and their aggregates up to 500 μm (Fig. 1 A). The sample of PZQ after processing in a planetary mill for 5 min at 20 g consists of particles mainly with a size of 50– 100 μm (Fig. 1B) and the sample after processing for 5 min at 40 g have the aggregates with sizes of 150–200 μm (Fig. 1C). Probably, an increase in the size of the aggregates in the latter case is associated with partial melting of the substance and subsequent sticking. The initial Na_2_GA consists of spherical particles with a size of 5–50 μm (Fig. 1D). The particles of the initial substances were crushed to poly-disperse powder consisting of particles with an average size of 5–20 μm and their aggregates after joint mechanochemical processing of PZQ and Na_2_GA (Fig. 1E). It can be assumed that PZQ is not a highly crystalline substance based on broadening of the reflexes on the radiographs of original PZQ (Fig. 2).

Moreover, its enthalpy of melting is ~ 90 J/g. The type of radiograph does not change significantly, the enthalpy of melting slightly decreases to 85 J/g after processing of PZQ in a planetary mill at 20 g for 5 minutes. The intensity of the reflexes on radiographs decreases after processing of PZQ in a planetary mill at 40 g for 5 min, but complete amorphization does not occur; the enthalpy of melting is 80 J/g. X-ray analysis and thermal analysis of supramolecular complex of PZQ with Na_2_GA indicated a loss of crystallinity of the initial components up to the formation of amorphous phases.

**Fig. 3:**
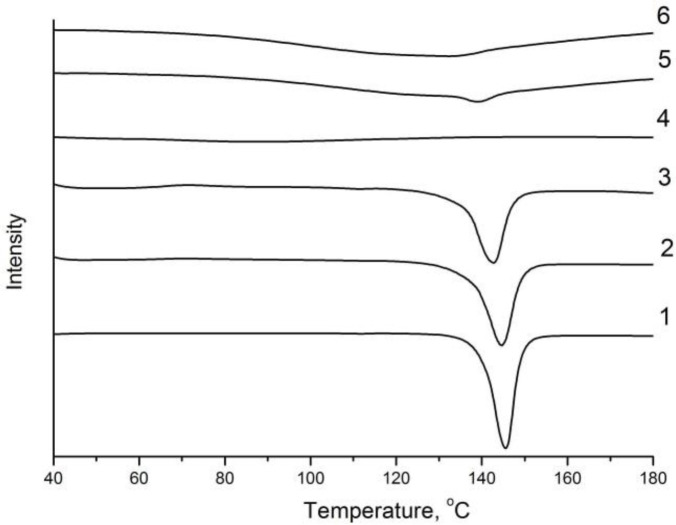
Differential scanning calorimetric thermal curves of: 1. praziquantel; 2. praziquantel processed in AGO-2 mill (20g, 5 min.); 3. praziquantel processed in AGO-2 mill (40g, 5 min.); 4. Na_2_GA; 5. physical mixture of praziquantel/Na_2_GA 1/10; 6. praziquantel/Na_2_GA 1/10 processed in ball drum mill for 4 hours

At the same time PZQ is distributed in the excess of the amorphous matrix of Na_2_GA, thereby greatly facilitating the formation of the guest-host system (Fig. 2–3).

In all cases, an increase in the solubility of PZQ after mechanochemical processing can be observed, which indicates the formation of intermolecular complexes of PZQ and Na_2_GA. The table 1 shows the maximum achieved solubility values. The HPLC data also confirmed that no degradation or chemical interactions involving molecules of PZQ have occurred.

**Table 1:** The solubility of PZQ and its compositions

***The composition of the sample, the weight ratio, the production method***	***Concentration of PZQ in water, g/l***	***The increase in solubility, n***	***The content of PZQ, % of theoretically***	***pH***
Initial PZQ	0.234	-		7.2
PZQ/Na_2_GA 1/10 physical mixture	0,566	1.42	100	5.9
PZQ/Na_2_GA 1/5 м/ch	0.557	1.94	99.9	5.8
PZQ/Na_2_GA1/10 м/ch	0.687	2.94	99.6	6.0
PZQ/Na_2_GA1/20 м/ch	0.817	3.49	100.0	6.2

**Fig. 4:**
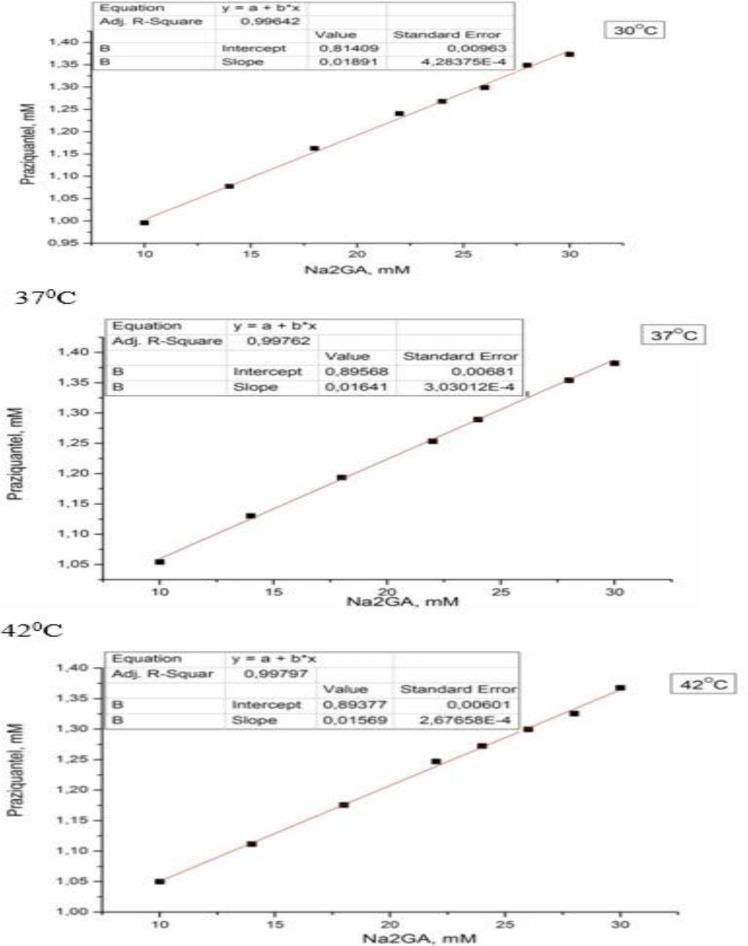
Phase diagrams of the solubility of PZQ/Na_2_GA 1/10 (mass) compositions in aqueous solutions at different temperatures

The phase diagrams of the solubility of PZQ from the compositions at different temperatures (Fig. 4, Table 2) were evaluated to prove intermolecular interactions in the aqueous solutions of PZQ with Na_2_GA and to estimate the stoichiometric and thermodynamic parameters of the inclusion of PZQ molecules into GA micelles. The resulting diagrams correspond to А_L_-type, which implies the formation of a complex with the stoichiometric ratio PZQ/Na_2_GA micelle 1/1.

**Table 2:** Thermodynamic parameters of the inclusion of PZQ molecules into micelles in Na_2_GA solution (mean±SE, n=3)

***T, °C***	***K, M^−1^***	***ΔG, kJ/mol***
+30	23.7±0.6	−8.0±0.1
+37	18.6±0.4	−7.5±0.1
+42	17.8±0.3	−7.6±0.1

**Fig. 5:**
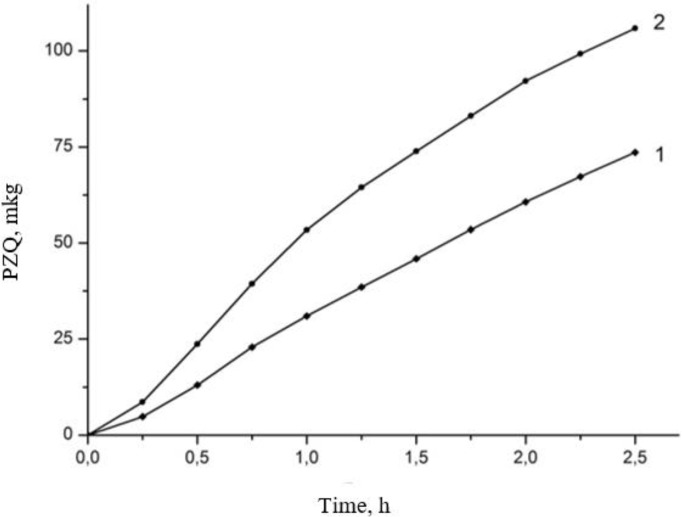
Membrane permeability of praziquantel. 1 – initial PZQ, 2 - PZQ/Na_2_GA 1/10

The results of the measurement of trans-membrane transfer are shown in Fig. 5. Evidently the rate of diffusion of PZQ molecules from its composition with Na_2_GA is much higher than that for initial PZQ substance. A substantial nearly 2-fold (for experiment duration 1 h) acceleration of transmembrane transfer from the solution of the complex was observed.

The sample selected on the basis of the best physicochemical properties – the supramolecular complex of PZQ with Na_2_GA (1:10) was tested on the anthelmintic efficacy on the laboratory model of *H. nana* infection of mice and *M. expansa* infection of sheep.

This study demonstrated 100 % efficacy of supramolecular complex of PZQ at the dose of 3 mg/kg (Table 3) in *H. nana* infection of mice while the unmodified PZQ showed 27.19 % efficacy. At necropsy, 10 adults of *H. nana* were found in the small intestine of mice of control group indicating the presence of an established infection. Statistically all the treatment groups had significantly fewer number of *H. nana* (*P* < 0.001) compared with the control group. Thus, on average, 0.032 and 0.264 *H. nana* (geometric mean) were detected in the small intestines of mice after administration of supramolecular complex of PZQ at doses of 2.0 and 1.0 mg/kg respectively. The PZQ:Na_2_GA1/10 physical mixture revealed no anthelmintic efficacy.

**Table 3:** *Hymenolepis nana* geometric counts, efficacy and statistical significance for treatment group (n = 10)

***Group of animals***	***Supramolecular complex and its composition***	***Dose, mg of active substance/kg of body weight***	***Geometric mean of worms***	***Efficacy^a^ (%)***	***P-Value***
Control	Placebo	–	24.423	NA	NA
Treatment	PZQ:Na_2_GA	3.0	0	100	< 0.0001^*^
Treatment	PZQ:Na_2_GA	2.0	0.032	99.99	< 0.0001^*^
Treatment	PZQ:Na_2_GA	1.0	0.264	98.92	< 0.001^*^
Treatment	PZQ substance	1.0	17.783	27.19	>0.05^*^
Treatment	PZQ:Na_2_GA1/10 physical mixture	2.0	16.5	32.44	>0.05^*^

a Percent efficacy based on geometric means

* Statistically significant at *P* ≤ 0.05 when geometric means were compared to placebo

The study showed 100 % efficacy of supra-molecular complex of PZQ against *M. expansa* infection of sheep at the dose of 3.0 mg/kg of b/w (Table 4). Worms of *M. expansa* were found in the small intestine of sheep after administration of supramolecular complex of PZQ at doses of 2.0 and 1.0 mg/kg of b/w in the amount of 0.003 and 0.064 parasites per animal. The efficacy was 99.91 and 98.18 % respectively based on the geometric mean counts. At necropsy sheep of all the treatment groups had significantly fewer *M. expansa* worms (*P*<0.0001) compared with the control group. The unmodified PZQ showed 36.64 % efficacy against *M. expansa* infection at the dose of 1 mg/kg.

**Table 4:** *Moniesia expansa* geometric counts, efficacy and statistical significance for treatment group

***Group of animals***	***Supramolecular complex and its composition***	***Dose, mg of active substance/kg of body weight***	***Number of sheep in group***	***Geometric mean of worms***	***Efficacy^a^ (%)***	***P-Value***
Control	Placebo	–	10	3.524	NA	NA
Treatment	PZQ:Na_2_GA	3.0	10	0	100	< 0.0001^*^
Treatment	PZQ:Na_2_GA	2.0	11	0.003	99.91	< 0.001^*^
Treatment	PZQ:Na_2_GA	1.0	12	0.064	98.18	< 0.001^*^
Treatment	PZQ substance	1.0	11	2.233	36.64	>0.05^*^

a Percent efficacy based on geometric means

* Statistically significant at *P* ≤ 0.05 when geometric means were compared to placebo

On average, 3.52 *M. expansa* worms were found in the small intestine of sheep in the control group that demonstrated an established infection of sheep in current study.

## Discussion

PZQ is the main drug for the treatment of humans and animals’ opisthorchiasis, clonorchosis, schistosomiasis, echinococcosis, cysticercosis, hymenolepiasis and other intestinal cestodosis (2, 24). Insufficient efficacy of PZQ in some helminthosis, including schistosomiasis, opisthorchiasis is caused by its low solubility and rapid metabolism in the liver with forming inactive compounds (25, 26). In this regard, the drug is used in an increased dose and in several ways to achieve maximum efficacy. Polyvinylpyrrolidone (27), polysaccharides (11,15), GA, Na_2_GA (6), are also used as a means for targeted delivery of anthelmintics. GA is able to form self-associates - micelles in aqueous solution because of the amphiphilic structure (7). In this case, poorly soluble molecules of medicinal substances are included in the structures of micelles and thereby increase their concentration in solution. The best solution in this respect is Na_2_GA as a “vehicle” (28).

Our studies of physicochemical properties confirmed an increase in the solubility of the supramolecular complex of PZQ with Na_2_GA in comparison with basic PZQ and the loss of its crystallinity up to the formation of amorphous phases. Solubility changed depending on the mass ratio of the components, the method of obtaining the compositions and time of mechanochemical processing. Since an increase in the proportion of PZQ leads to decrease in solubility and an increase in the proportion of Na_2_GA overloads the dosage form for oral administration. Therefore, 1/10 mass ratio of PZQ/Na_2_GA was optimal for further studies and had a good solubility index. Thermal and X-ray analyses showed that reflexes and melting peaks that are characteristic of the initial crystalline phase of PZQ have disappeared after mechanochemical processing. It can be assumed that molecules of PZQ are distributed in the amorphous Na_2_GA matrix, thereby greatly facilitating the formation of the guest-host system, where the guest is the molecules of PZQ and the host is the Na_2_GA carrier. This distribution significantly changes the properties of the drug and facilitates its release and transfer through biological membranes (29). The micrographs obtained by electronic scanning microscopy revealed that reduction of the size of particles of supramolecular complex of PZQ. The occurrence of the intermolecular interaction of PZQ with the micelles of GA in solution was demonstrated by means of phase solubility. Investigation of the permeability of PZQ through the artificial membrane by PAMPA assay demonstrated that the rate of PZQ molecule diffusion from its composition with Na_2_GA is much higher than that of initial PZQ.

In the present study the efficacy of the supramolecular complex of PZQ with Na_2_GA was three times higher than the efficacy of the substance of PZQ in the experiment carried out on white mice experimentally infected with *H. nana* and sheep naturally infected with moniezia at the dose of 1 mg/kg of b/w.

## Conclusion

Joint mechanochemical processing of the PZQ substance and Na_2_GA led to reduction of particle sizes, increased solubility, amorphization of substance, incorporating it with micelles of GA and high anthelmintic efficacy in reduced dose. The supramolecular complex of praziquantel was found to be a perspective anthelminthic with enhanced pharmacological activity that needs further research.

## Ethical considerations

Ethical issues (Including plagiarism, informed consent, misconduct, data fabrication and/or falsification, double publication and/or submission, redundancy, etc.) have been completely observed by the authors.
